# The Human Gene Mutation Database: towards a comprehensive repository of inherited mutation data for medical research, genetic diagnosis and next-generation sequencing studies

**DOI:** 10.1007/s00439-017-1779-6

**Published:** 2017-03-27

**Authors:** Peter D. Stenson, Matthew Mort, Edward V. Ball, Katy Evans, Matthew Hayden, Sally Heywood, Michelle Hussain, Andrew D. Phillips, David N. Cooper

**Affiliations:** School of Medicine, Institute of Medical Genetics, Cardiff University, Heath Park, Cardiff, CF14 4XN UK

## Abstract

The Human Gene Mutation Database (HGMD^®^) constitutes a comprehensive collection of published germline mutations in nuclear genes that underlie, or are closely associated with human inherited disease. At the time of writing (March 2017), the database contained in excess of 203,000 different gene lesions identified in over 8000 genes manually curated from over 2600 journals. With new mutation entries currently accumulating at a rate exceeding 17,000 per annum, HGMD represents de facto the central unified gene/disease-oriented repository of heritable mutations causing human genetic disease used worldwide by researchers, clinicians, diagnostic laboratories and genetic counsellors, and is an essential tool for the annotation of next-generation sequencing data. The public version of HGMD (http://www.hgmd.org) is freely available to registered users from academic institutions and non-profit organisations whilst the subscription version (HGMD Professional) is available to academic, clinical and commercial users under license via QIAGEN Inc.

## Introduction

The Human Gene Mutation Database (HGMD^®^) represents an attempt to collate all known gene lesions underlying human inherited disease together with disease-associated/functional polymorphisms published in the peer-reviewed literature. The mutation data catalogued by HGMD (summarised by mutation type) are shown in Table 1.

**Table 1:** Numbers of different mutations by mutation type present in HGMD Professional release 2017.1 and the publicly available version of the database (March 31st 2017)

Mutation type	Numbers of mutations		
	HGMD Professional 2017.1		Publicly available
	Total *(disease*-*associated/functional polymorphism sub*-*total*)	With chromosomal coordinates and VCF data (GRCh38/hg38)	
Missense substitutions	92,331 *(5132)*	91,671	62,759
Nonsense substitutions	22,372 *(333)*	22,376	15,642
Splicing substitutions (intronic and exonic)	18,386 *(632)*	18,083	13,087
Regulatory substitutions (exonic, intronic, 5′- and 3′-untranslated regions)	3801 *(2499)*	3717	2764
Micro-deletions ≤20 bp	30,169 *(292)*	29,540	21,744
Micro-insertions/duplications ≤20 bp	12,557 *(175)*	12,227	8975
Micro-indels ≤ 20 bp	2866 *(59)*	2770	2100
Gross deletions >20 bp	15,272 *(147)*	0	10,337
Gross insertions/duplications >20 bp	3767 *(84)*	0	2389
Complex rearrangements (including inversions, translocations and complex indels)	1857 *(117)*	0	1417
Repeat variations	507 *(306)*	0	421
Totals	203,885 *(9776)*	180,386	141,635

HGMD has never sought to include either somatic or mitochondrial mutations, which are well covered by COSMIC (Forbes et al. 2015) and MitoMap (Lott et al. 2013), respectively. Nor does HGMD attempt to provide comprehensive coverage of pharmacological variants (except for those variants where evidence supporting a functional impairment has been provided); PharmGKB (https://www.pharmgkb.org/; Thorn et al. 2013) is a more comprehensive resource for these data. Finally, HGMD is not intended to be a general genetic variation database; users interested in such variants should visit dbSNP (http://www.ncbi.nlm.nih.gov/SNP/; Sherry et al. 2001), the NHLBI Exome Variant Server (http://evs.gs.washington.edu/EVS/) or the Exome Aggregation Consortium (ExAC; http://exac.broadinstitute.org/; Lek et al. 2016).

HGMD was originally established in 1996 for the scientific study of mutational mechanisms in human genes believed to cause inherited disease (Cooper et al. 2010; Stenson et al. 2014). However, over the last 20 years it has acquired a much broader utility as the central unified repository for disease-related functional genetic variation in the germline. It is now routinely accessed and utilised by next-generation sequencing (NGS) project researchers, human molecular geneticists, molecular biologists, clinicians and genetic counsellors as well as by those specialising in biopharmaceuticals, bioinformatics and personalised genomics.

The public version of HGMD (http://www.hgmd.org) is freely available to registered users from academic institutions/non-profit organisations. This version is, however, maintained in a basic form that is only updated twice annually, is permanently a minimum of 3.5 years out of date, and does not contain any of the additional annotations or extra features present in HGMD Professional (see below). The Professional version is available to both commercial and academic/non-profit users via subscription from QIAGEN (https://www.qiagenbioinformatics.com/) as either an online or a locally installed/downloadable version that is updated quarterly and includes a variety of additional annotations and extra features, such as GRCh38/hg38 and GRCh37/hg19 genomic chromosomal coordinates, HGVS nomenclature, Variant Call Format (VCF), additional literature reports, advanced search features, conservation data and functional predictions.

## Source of mutation data

**Fig. 1:**
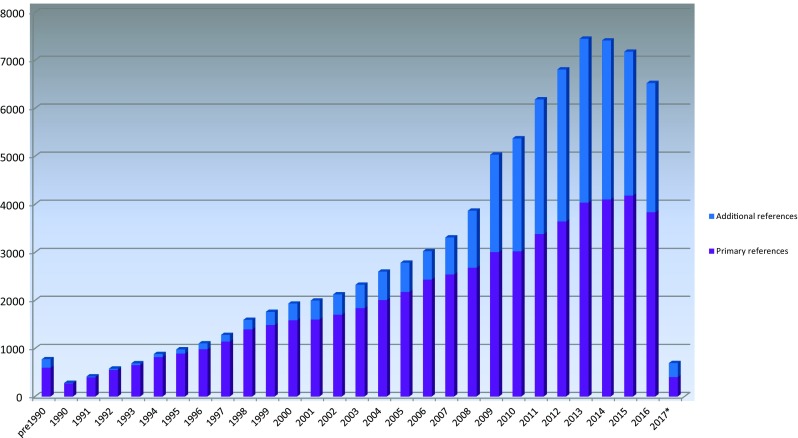
Annual numbers of cited literature references added to HGMD. *2017 figures not yet complete

All HGMD mutation data have been obtained from the scientific literature and are manually curated on an ongoing basis. Identification of relevant literature reports is carried out via a combination of manual journal screening and automated text mining. The database currently contains >203,000 mutation entries obtained from over 57,000 primary literature reports (supported by 29,000 additional literature reports), which were published in more than 2600 different journals. The number of articles screened (both for novel mutations and additional annotations) appears to have reached a plateau, (Fig. 1); however, the number of mutations reported (per reference) continues to increase steadily. It is likely that the continuing development of high-throughput NGS methods will lead to an increased rate of deposition of disease-associated genetic variants in the published literature.

## Classes of variant listed in HGMD

**Fig. 2:**
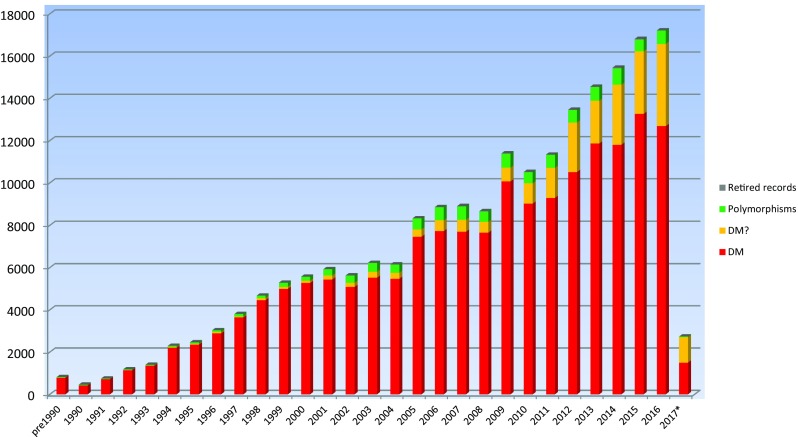
Annual mutation totals subdivided by variant class. *2017 figures not yet complete

There are six different classes of variant listed in HGMD (Fig. 2). Disease-causing mutations (DM) are entered into HGMD where the authors of the corresponding report(s) have established that the reported mutation(s) are involved (or very likely to be involved) in conferring the associated clinical phenotype upon the individuals concerned. The DM classification may, however, also appear with a question mark (DM?), denoting a probable/possible pathological mutation, reported as likely to be disease causing in the corresponding report, but where (i) the author has indicated that there may be some degree of doubt or uncertainty; (ii) the HGMD curators believe greater interpretational caution is warranted, or (iii) subsequent evidence has appeared in the literature which has called the initial putatively deleterious nature of the variant into question (e.g. a negative functional, case–control or population-scale sequencing study). The DM and DM? variant classes may include mutations that are believed to contribute to disease susceptibility in a multi-factorial manner (e.g. autism or schizophrenia), exhibit complex polygenic inheritance or possess an environmental trigger component to their pathogenicity. It can be seen from Fig. 2 that the proportion of reported mutations belonging to the DM? category has steadily increased over the last decade; we speculate that this is because authors, journal editors and referees (also database curators!) alike have become much more cautious than they used to be in ascribing pathogenicity to the putatively disease-associated variants that have been identified. This increase in caution appears to closely coincide with the advent of NGS and the consequent deluge of genetic variants that must be filtered and prioritised.

Three categories of polymorphism are included in the database (combined into ‘polymorphisms’ in Fig. 2). Disease-associated polymorphisms (DP) are entered into HGMD where there is evidence for a significant association with a disease/clinical phenotype along with additional evidence that the polymorphism is itself likely to be of functional relevance (e.g. as a consequence of genic location, evolutionary conservation, transcription factor binding potential, etc.), although there may be no direct evidence (e.g. from an expression study) for a functional effect. The functional polymorphisms (FP) class includes those sequence changes for which a direct functional effect has been demonstrated (e.g. by means of an in vitro reporter gene assay or alternatively by protein structure, function or expression studies), but with no disease association reported as yet. Disease-associated polymorphisms with supporting functional evidence (DFP) must meet both of the above criteria in that the polymorphism should not only have been reported to be significantly associated with disease, but should also display direct evidence of being of functional relevance. The polymorphism data present in HGMD should be viewed with a degree of caution owing to (i) the possibility that an observed disease association may be simply due to a linkage disequilibrium effect and (ii) the fact that in vitro studies are not invariably accurate indicators of in vivo functionality (Cirulli and Goldstein 2007; Dimas et al. 2009). Retired records (R) are variants that have been removed from HGMD if found to have been erroneously included ab initio, or if the variant has been subject to retraction/correction in the literature resulting in the record becoming obsolete, merged or otherwise invalid.

The various HGMD variant classes described above should not be cross-correlated with the ‘benign to pathogenic’ 5-point classification system adopted by the ACMG consortium (Green et al. 2013). Although, by their very nature, there will be some overlap, these two classification systems are not directly interchangeable. The primary purpose of the ACMG guidelines appears to be to minimise false positives in a clinical setting, whereas HGMD aims to include mutation data based on the cogency and credibility of the associated literature, with a curation policy that opts to minimise false negatives by being broadly inclusive, whilst attempting to highlight potential false positives to users (e.g. via an allele frequency flag). Attempting to cross-correlate the two classification systems (e.g. by automatically considering HGMD DM to be equivalent to ACMG class 5) is likely to be potentially misleading at best, and may well lead to users drawing incorrect or inappropriate conclusions (Pinard et al. 2016).

Polymorphic copy number variations (CNVs) represent an important subset of potentially functional disease-associated variation (Mikhail 2014; Usher and McCarroll, 2015). While HGMD does not wish to replicate the excellent curatorial work of other resources (e.g. the Database of Genomic Variants http://dgv.tcag.ca/dgv/app/home, DECIPHER http://decipher.sanger.ac.uk/ and Copy Number Variation in Disease http://202.97.205.78/CNVD/), we do include such variants where they fulfil certain criteria. HGMD will include such variants if they have been shown to be of functional significance, associated with disease, and involve a single characterised gene or small group of genes that have been directly implicated in the disease association. Such variants would then be entered into the database under one of the above-mentioned polymorphism categories, depending upon the supporting evidence provided by the authors of the article in question.

The HGMD curators have adopted a policy of continual reassessment of the curated content within the database. If and when newly published information relevant to a specific mutation entry becomes available (e.g. additional case reports or alternate clinical or laboratory phenotypes, population frequency data or functional studies), the mutation entry may be revised or re-classified. Where new information becomes available which suggests that a given disease-causing mutation (DM) is likely to be of questionable pathological relevance or even a neutral polymorphism (on the basis of additional case reports, genome/population screening studies, negative case–control studies, etc.), it may be flagged with a question mark (DM?), re-categorised under one of the categories of polymorphism, or retired from the database altogether (R) if it turns out to have been erroneously included ab initio. The HGMD curators re-categorised or retired over 800 variants in 2015 with almost 26,000 existing records having at least one relevant additional reference added in the same year. Users of HGMD may utilise a feedback/comments function in order to inform the HGMD curators of relevant new or missing information, or to request the correction, recategorisation or removal of a listed variant.

Zygosity information (i.e. heterozygous, homozygous or compound heterozygous) for individual mutations in HGMD has not been recorded. Reasons for this include (i) the fact that this information is not always unequivocally provided in the corresponding literature reference; (ii) the possibility that a given mutation may be pathogenic irrespective of the zygosity in which it is found; (iii) the clinical consequences of zygosity may often be modified by other genetic variants either in *cis* or in *trans*; (iv) digenic or polygenic inheritance of other pathogenic variants or disease modifiers and (v) variable or reduced penetrance which ensures that the genotype is not invariably predictive of the clinical phenotype (Cooper et al. 2013). Sometimes the same mutation may be present in the heterozygous, compound heterozygous or homozygous states in different patients; in such cases, information on zygosity may not be easy to provide and may be even more difficult to interpret. Thus, information pertaining to zygosity would not always be helpful or informative with regard to ascertaining or predicting the clinical phenotype, and indeed might even prove inaccurate or misleading.

HGMD users should not assume that just because a sequence variant is labelled “DM”, it automatically follows that it is known or believed to be pathogenic in all individuals harbouring it (i.e. that the variant exhibits 100% penetrance). Indeed, many “disease-causing mutations” display reduced or variable penetrance for a variety of different reasons (reviewed by Cooper et al. 2013). Further, population sequencing programmes (such as the 1000 Genomes Project and ExAC) are now identifying considerable numbers of “DM” mutations in apparently healthy individuals (MacArthur et al. 2012; Xue et al. 2012; Lek et al. 2016). Such lesions should not be regarded automatically as being clinically irrelevant, even when they occur with significant frequency, because it is quite possible that these mutations either represent low-penetrance, mild or late onset, or more complex disease susceptibility alleles, as opposed to neutral variants (Cooper et al. 2013), or alternatively reside within transcripts that exhibit a degree of translational plasticity (Jagannathan and Bradley 2016).

**Fig. 3:**
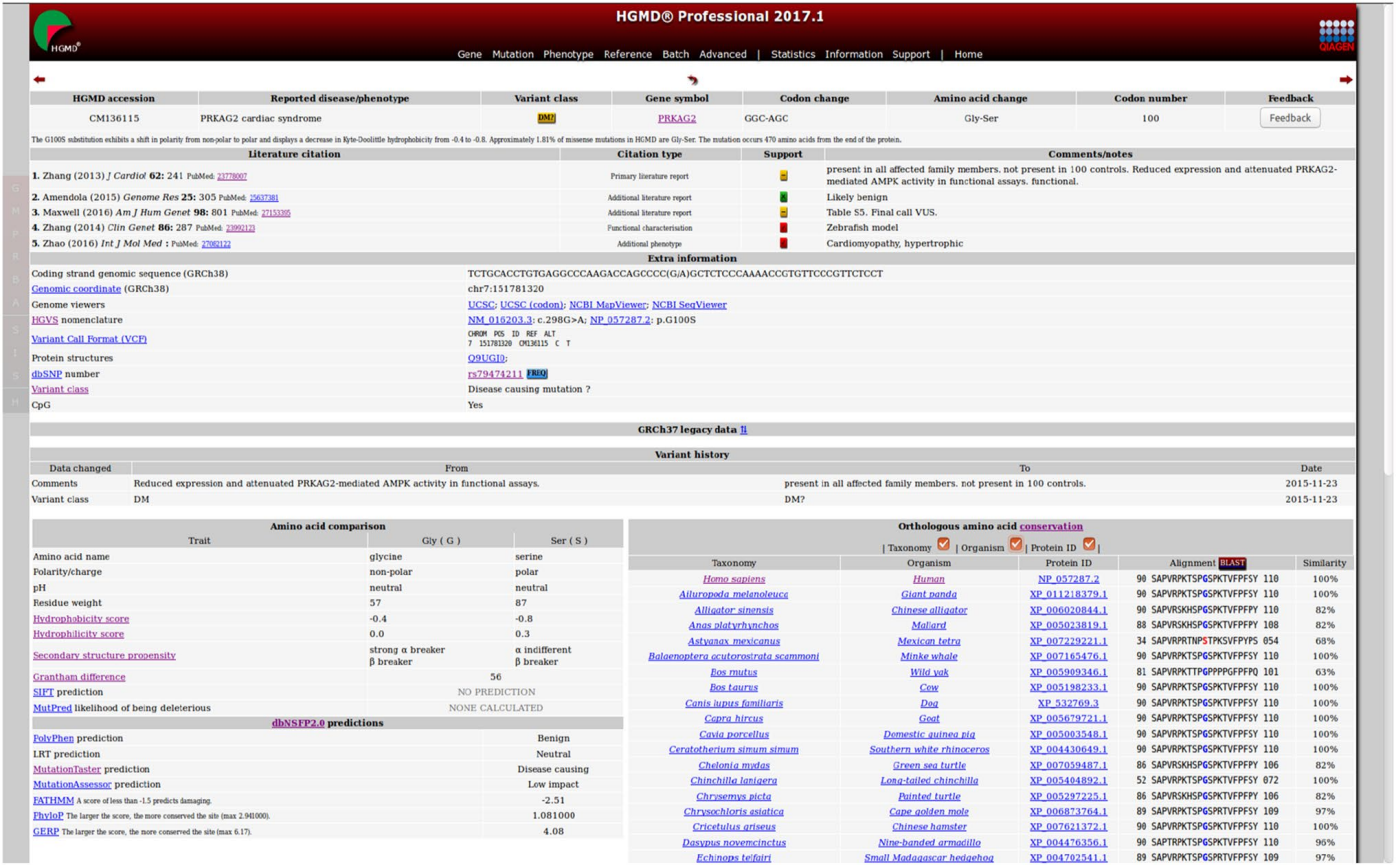
Example of an HGMD Professional entry

It is HGMD curation policy to err on the side of inclusion and enter a variant into the database even if its pathological relevance may be questionable (while indicating this fact to our users wherever feasible), rather than run the risk of inadvertently excluding a variant that may be directly (or indirectly) relevant to disease. We have taken several different steps to highlight such equivocation in HGMD, viz. the DM? variant class, a dbSNP 1000 Genomes frequency flag (to highlight those HGMD variants that are also present in dbSNP, with allele frequency information included; see below) and the provision of additional literature citations which either support or cast doubt upon the pathogenicity of a particular variant (Fig. 3). This latter point is particularly pertinent in the clinical setting, where a greater burden of proof may be required as a prerequisite for use in diagnostic and predictive medicine, and when considering the return of incidental findings to patients after testing (Green et al. 2012, 2013; Ng et al. 2013; Gonsalves et al. 2013; Dewey et al. 2014; Tabor et al. 2014; Gambin et al. 2015; Jurgens et al. 2015).

Additional literature references are an important source of contextual information, and play a vital role in querying or confirming the pathogenicity of HGMD variants. Types of additional reference include functional studies, additional case reports, additional phenotypes and population case–control studies. The number of additional references in HGMD has grown steadily as a proportion of the total number of references and accounts for approximately 30–40% of the number of literature references screened and entered into HGMD over the last 3–5 years (Fig. 1). The number of literature references reporting novel variants appears to have reached a plateau over the last few years; however, the number of variants being reported per reference is still increasing, from 2.5 mutations per reference in the 1990s to over 4.0 in the last two years. We expect this trend to continue as ever larger numbers of patient population-scale sequencing studies are completed and published (Ellingford et al. 2016; Susswein et al. 2016; Lopes et al. 2015).

## HGMD Professional

HGMD Professional serves as the subscription version of HGMD, and is available to both commercial and academic customers under license from QIAGEN Inc. HGMD Professional allows access to up-to-date mutation data with a quarterly release cycle; this version is therefore essential for checking the novelty of newly found mutations. HGMD Professional contains many features not available in the public version. More powerful search tools in the form of an expanded search engine with full text Boolean searching are provided. A batch search mode has been developed to allow users to query HGMD using gene (e.g. OMIM IDs, Entrez IDs), variant (e.g. dbSNP IDs, chromosomal coordinates, VCF format) and dataset (e.g. PubMed ID) oriented lists. Users can employ these tools to perform additional searches for gene-specific (e.g. chromosomal locations, gene names/aliases and gene ontology), mutation-specific (e.g. chromosomal coordinates, HGVS nomenclature, dbSNP ID) or citation-specific (e.g. first author, publication year, PubMed ID) information. Chromosomal coordinates (hg19/hg38) and HGVS nomenclature are provided for the vast majority of our nucleotide substitutions (99.8% coverage) and other micro-lesions (97.6% coverage). Provision of consistently accurate mutation descriptions is especially important in the era of NGS sequencing (Yen et al. 2017) and has helped to make HGMD an invaluable tool for the analysis of population-scale NGS datasets such as the 1000 Genomes Project (1000 Genomes Project Consortium 2015) and ExAC (Lek et al. 2016). Additional information is also provided on a mutation-specific basis (see Fig. 3) including curatorial comments (for example, if the mutation data presented in the original publication required in-house correction or author clarification [5–10% of all entries], or if the clinical phenotype is associated with a more complex, i.e. digenic or in-*cis* inheritance pattern), additional reports comprising functional characterisation, further phenotypic information, comparative biochemical parameters, evolutionary conservation and SIFT (Sim et al. 2012) and MutPred (Li et al. 2009) pathogenicity predictions. More recently, the functional predictions and nucleotide conservation data from dbNSFP2.0 (Liu et al. 2013), a database of all potential non-synonymous single-nucleotide variants in the human genome, have been included. These additional annotations are updated on a regular basis.

**Fig. 4:**
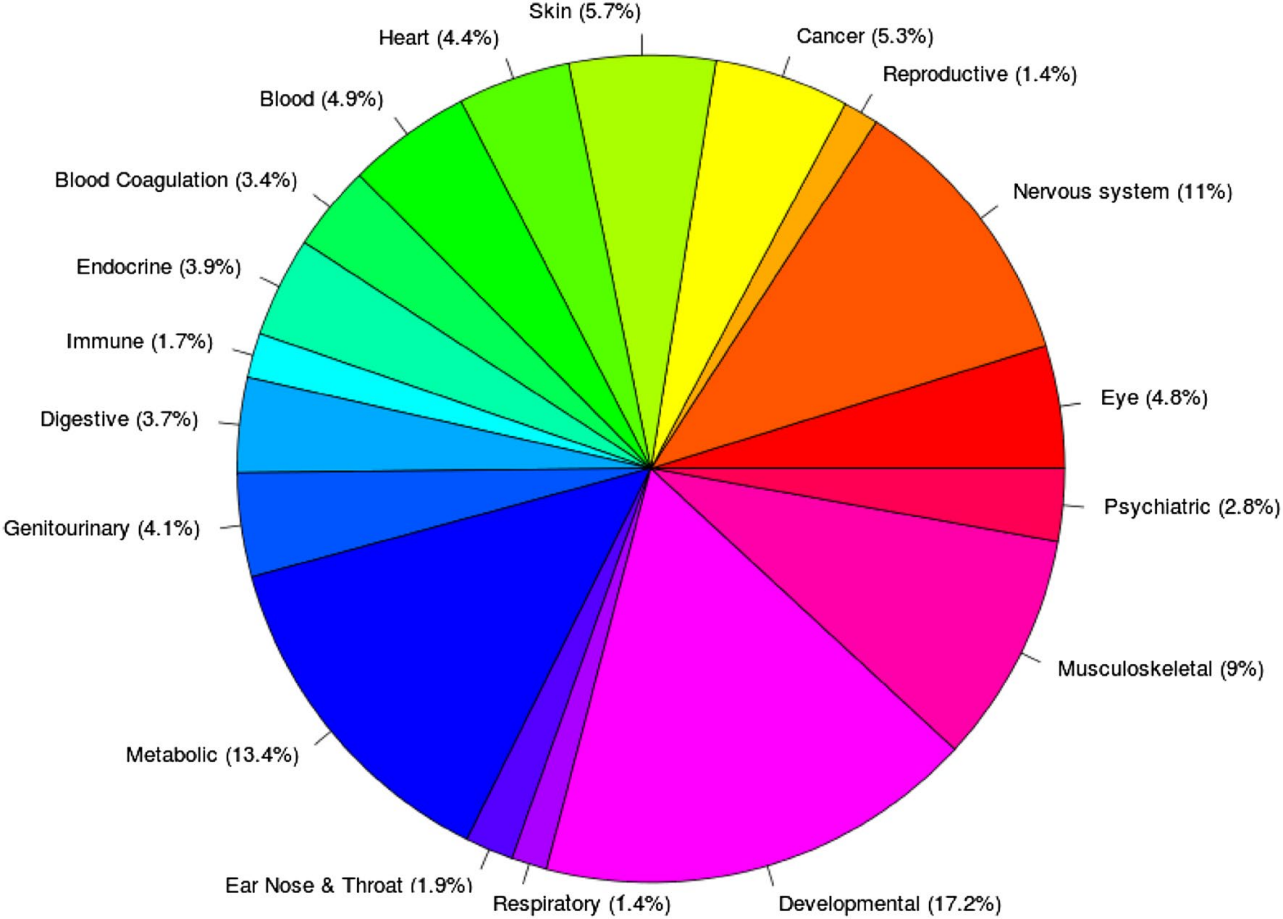
Overview of UMLS high-level disease concept mappings present in HGMD

HGMD clinical phenotypes have been annotated against the Unified Medical Language System (UMLS) using a combination of manual curation and natural language processing. The UMLS is a compilation of biomedical ontologies and vocabularies catalogued into a single resource (e.g. OMIM phenotype data, Medical Subject Headings (MeSH) and other disease ontologies), and may be found at http://www.nlm.nih.gov/research/umls/. HGMD phenotype data have been mapped to approximately 18 different UMLS high-level concepts (Fig. 4). These UMLS mappings provide users with a more accurate and expanded phenotype search. Thus, searches using alternative disease names should return the same result-set, e.g. a search for “breast cancer” should yield identical results to a search for “malignant neoplasm of breast”. In addition, utilising the UMLS allows for powerful semantic searching (e.g. searches for all mutations linked to “blood disorders” or “immune disorders”). The UMLS ontology mappings have been utilised in a variety of different NGS sequencing studies (see below).

Another feature involves the highlighting of HGMD entries where the pathogenicity of the variant may have been cast into doubt by virtue of its high allele frequency. HGMD Professional displays a frequency flag when a listed variant is to be found in dbSNP, and population frequency data from the 1000 Genomes Project are provided. In addition, HGMD will soon include allele frequencies derived from the more recent ExAC study (Lek et al. 2016). As well as searching and viewing mutation data, users of HGMD Professional may utilise a feedback facility to submit corrections to the database curators or to request additional features (see Fig. 3 to view a sample HGMD Professional variant entry).

HGMD Professional also includes an Advanced Search facility to enhance mutation searching, viewing and retrieval. Datasets may be combined (for example, micro-deletions, micro-insertions and indels) to enable powerful searching across comparable types of mutation. A variety of search parameters are available, including functional features [e.g. in vitro and/or in silico characterised transcription factor binding sites, post-translational modifications, microRNA binding sites, upstream open reading frames (ORFs), and catalytic residues] to search for the gain or loss of a specific feature as a consequence of mutation; type of amino acid substitution; nucleotide substitution; size and/or sequence composition of micro-deletions, micro-insertions or indels; pre- or user-defined sequence motifs (both those created and those abolished by the mutation in question); dbSNP number; keywords found in the article title or abstract. The Advanced Search also includes a batch mode termed “Mutation Mart” to query HGMD via multiple identifiers including dbSNP, Entrez gene (http://www.ncbi.nlm.nih.gov/gene) and PubMed. HGMD Professional is available to subscribers either as an online-only package or in downloadable form enabling users to incorporate HGMD data into their local variant analysis pipelines (https://www.qiagenbioinformatics.com/products/human-gene-mutation-database/).

## Focus on NGS

HGMD data are available in VCF format allowing easy visualisation, for example by using the Integrative Genomics Viewer (Robinson et al. 2011), or incorporation into custom data analysis pipelines (Dorschner et al. 2013; Gambin et al. 2015; Johnston et al. 2015; Lek et al. 2016). This facility allows users to maximise their use of HGMD data in both a clinical diagnostic and research setting. The provision of disease UMLS concept mappings (including OMIM, SNOMED, MeSH and HPO) also greatly enhances both the web-based HGMD search facility and the downloadable package, allowing the stratification of variants according to recognised disease concepts.

When using HGMD Professional to annotate large NGS datasets, and depending on the context (e.g. an inherited disease screen), it is often useful to annotate the dataset with a subset of HGMD variants (e.g. those which fall into the DM and DM? categories). Any variants found concurrently in this subset and the dataset being tested may then be further prioritised by variant class; hence, DM variants could be ranked higher than DM? variants if so desired. We have plans to introduce a literature-based variant scoring system to allow NGS researchers and clinicians to improve their prioritisation of DM/DM? variants found in their result sets. This system will annotate additional references as being supportive, neutral or not supportive of the inclusion of the variant in HGMD, thereby allowing users to rank those variants that possess additional supporting literature evidence (e.g. those with a published functional study) more highly, in addition to de-prioritising variants that have additional literature evidence questioning their pathological relevance. This new information will be available in both the online and download versions of the next release of HGMD Professional (see Fig. 3).

One of the problems encountered by NGS researchers and clinicians is the mis-annotation of variants as pathogenic or disease-causing. A small number of literature reports have been published where common variants have not being properly filtered out at an early stage, thereby increasing the number of mis-categorised variants appearing in the literature. HGMD has instigated plans to mitigate this problem, including the pre-screening of entries against the population frequency data present in ExAC (in progress) and the introduction of a literature-based scoring system (see above).

## Other variant databases

Several other databases are available that attempt to record disease-causing or disease-associated (i.e. pathogenic) variation. These include the Online Mendelian Inheritance in Man, OMIM (http://www.omim.org/; Amberger et al. 2015), ClinVar (http://www.ncbi.nlm.nih.gov/clinvar/; Landrum et al. 2016), dbSNP (http://www.ncbi.nlm.nih.gov/SNP/; Sherry et al. 2001), LOVD (http://grenada.lumc.nl/LSDB_list/lsdbs; Fokkema et al. 2011) and a variety of locus-specific mutation databases (LSDBs) (http://www.hgvs.org/dblist/glsdb.html). OMIM does not provide statistics for allelic variants on its website; however, 25,115 germline OMIM variants appear to have been added to ClinVar, which itself currently contains a total of 53,211 pathogenic or likely pathogenic germline variants, whereas dbSNP contains 49,675 pathogenic or likely pathogenic clinically significant variants (all databases accessed December 30th 2016). In comparison, HGMD currently contains 193,904 DM and DM? variant entries in 6770 genes. Owing to the highly dispersed nature of the LSDBs and the potential for duplication between databases, accurate statistics with regard to like-for-like bona fide germline disease-causing (i.e. not merely neutral) variation is difficult to obtain. Since OMIM only records a limited number of variants deemed newsworthy per gene, and ClinVar still lacks depth (in terms of variant and literature coverage) and obtains a significant proportion (~40% of the above-mentioned total) of its pathogenic variant data via direct submission from clinical testing laboratories, HGMD is the only database of inherited human pathological variants that can claim to approach comprehensive coverage of the peer-reviewed literature (Peterson et al. 2013). Since both ClinVar and the LSDBs contain unpublished (i.e. non-peer reviewed) mutation data, the question has arisen as to whether HGMD should also include these data (Patrinos et al. 2012). However, both ClinVar and the LSDBs have encountered problems pertaining to data quality, submission, provenance and consent. A recent study (Abouelhoda et al. 2016) found that a higher proportion (1.1% vs. 0.59%) of variants in ClinVar required reclassification when compared to HGMD Professional (Abouelhoda et al. 2016, Table 1). The reclassification data presented by the authors of this study have already been incorporated into HGMD Professional. At present, however, it does not appear that any revisions have been made to ClinVar as a result of this study. Therefore, we have opted not to include data from these databases at this time.

## How HGMD is utilised

The registered users of the HGMD public website (>101,000 as of March 2017) performed more than 260,000 queries in 2016. HGMD data may not be downloaded in their entirety from the public website; however, data may be made available at the discretion of the curators for non-commercial research purposes. Potential collaborators who wish to access HGMD data in full are required to sign a confidentiality agreement.

HGMD data have been used to perform a series of meta-analyses on different types of gene mutation causing human inherited disease. These studies have helped to improve our understanding of mutational spectra and the molecular mechanisms underlying human inherited disease (Cooper et al. 2011). They have served to demonstrate not only that human gene mutation is an inherently non-random process, but also that the nature, location and frequency of different types of mutation are shaped in large part by the local DNA sequence environment (Cooper et al. 2011). Indeed, HGMD data have been instrumental in demonstrating that electron transfer reactions (Bacolla et al. 2013), base-pair flexibility (Bacolla et al. 2015) and non-B DNA forming sequences (Kamat et al. 2016) all contribute to sequence context-dependent mutagenesis causing inherited disease. HGMD mutation data were used to demonstrate that many in-frame pathogenic variations perturb protein–protein interactions (Das et al. 2014). HGMD mutations have also been used to demonstrate that proteins linked to autosomal dominant diseases exhibit more clustering of rare missense mutations than those linked to autosomal recessive diseases (Turner et al. 2015). Finally, HGMD mutations have been mapped to protein 3D structures in order to study the loss and gain of various types of functional attribute, thereby quantifying the impact of disease-causing amino acid substitutions on catalytic activity, metal binding, macromolecular binding, ligand binding, allosteric regulation and post-translational modification (Lugo-Martinez et al. 2016).

HGMD data have been used extensively in several international collaborative research projects including the Genotype-Tissue Expression (GTEx) project (Rivas et al. 2015), the ExAC project (Lek et al. 2016) and the 1000 Genomes project (Marth et al. 2011; MacArthur et al. 2012; 1000 Genomes Project Consortium 2015), where a surprising number of HGMD variants were found in apparently healthy individuals. They have also been used in the comparative analysis of orthologous sequences in model genomes including those of gorilla (Scally et al. 2012), mountain gorilla (Xue et al. 2015), cynomolgus and Chinese macaques (Yan et al. 2011), Rhesus macaque (Rhesus Macaque Genome Sequencing and Analysis Consortium 2007) and rat (Rat Genome Sequencing Project Consortium 2004), in which many apparently disease-causing mutations in human were found as wild-type (‘compensated mutations’) (Azevedo et al. 2015, 2016).

In a clinical setting, HGMD is widely utilised by many groups in ongoing NGS diagnostic (Bell et al. 2011; Johnston et al. 2012; Calvo et al. 2012; Makrythanasis et al. 2014; Karageorgos et al. 2015; Wilfert et al. 2016; Walsh et al. 2017) and human genome sequencing (Tong et al. 2010; Kim et al. 2009; Telenti et al. 2016) programmes. HGMD has also been used by a number of different groups to aid the development of a wide variety of post-NGS variant interpretation and exome prioritisation algorithms including MutPred (Li et al. 2009), MutPred Splice (Mort et al. 2014), PROVEAN (Choi et al. 2012), CAROL (Lopes et al. 2012), regSNPs (Teng et al. 2012), CRAVAT (Douville et al. 2013), NEST (Carter et al. 2013), FATHMM (Shihab et al. 2013), FATHMM-MKL (Shihab et al. 2015), PinPor (Zhang et al. 2014), MutationTaster2 (Schwarz et al. 2014), Phen-Gen (Javed et al. 2014), VEST-indel (Douville et al. 2016), Gene Damage Index (Itan et al. 2015), DDIG-in (Folkman et al. 2015), RSVP (Peterson et al. 2016), ExonImpact (Li et al. 2017), IntSplice (Shibata et al. 2016), snvForest (Wu et al. 2015), IMHOTEP (Knecht et al. 2017) and M-CAP (Jagadeesh et al. 2016). A list of some of the articles which have utilised HGMD data or expertise in their analyses can be found on the HGMD website (http://www.hgmd.cf.ac.uk/docs/articles.html).

## Data sharing

A limited HGMD data set, containing both chromosomal coordinates and HGMD identifiers, has been made available via academic data exchange programmes to the European Bioinformatics Institute (EBI)/Ensembl (Flicek et al. 2013) and the University of California, Santa Cruz (UCSC) (Meyer et al. 2013) and may be viewed in these projects’ respective genome browsers. Data from HGMD Professional have additionally been made available to subscribers of Ingenuity Variant Analysis™ (QIAGEN) and Alamut (Interactive Biosoftware), but are also accessible as part of the HGMD Professional stand-alone package (QIAGEN). Allowing free access to the bulk of the mutation data present in HGMD, while generating sufficient income to support maintenance and development via commercial distribution, represents a business model that is intended to maximise the availability of HGMD at the same time as ensuring its long-term sustainability. Although we are obliged to be prudent with regard to data sharing with public data repositories, we have always taken the view that making as much data publicly available as possible is generally beneficial to HGMD as well as to its users worldwide.

## Future plans

The provision of chromosomal coordinates (both GRCh37 and 38) for the vast majority of coding region micro-lesions in HGMD is now complete. Expanding this provision to include micro-lesions in non-coding regions and the gross (in progress) and complex lesion (where feasible) datasets is a high priority, We plan to add other commonly utilised NGS formats such as General Feature Format (GFF) (http://www.sanger.ac.uk/resources/software/gff/) and Browser Extensible Data (BED) format to complement the Variant Call Format (VCF) (Danecek et al. 2011) data currently available in HGMD Professional. The provision of allele frequency data from large-scale NGS projects such as ExAC (http://exac.broadinstitute.org/), more complete references (i.e. including article titles) and HGVS protein level descriptions for HGMD micro-lesions are also priorities. Provision of genomic reference sequences based on the NCBI RefSeqGene project (Pruitt et al. 2014), links to available protein structures and homology models, and expanding our coverage of secondary references (additional case reports and functional studies) are also regarded as priorities, as well as updating our set of functional predictions using the new dbNSFP v3.0 dataset (Liu et al. 2016).

HGMD provides the user with a unique resource that can be utilised not only to obtain evidence to support the pathological authenticity and/or novelty of detected gene lesions and to acquire an overview of the mutational spectra for specific genes, but also as a knowledgebase for use in the bioinformatics and whole genome screening projects that underpin personalised genomics, next-generation sequencing research and diagnostic medicine.
